# Post-diagnosis dietary insulinemic potential and survival outcomes among colorectal cancer patients

**DOI:** 10.1186/s12885-020-07288-0

**Published:** 2020-08-27

**Authors:** Fred K. Tabung, Anne Noonan, Dong Hoon Lee, Mingyang Song, Steven K. Clinton, Daniel Spakowicz, Kana Wu, En Cheng, Jeffrey A. Meyerhardt, Charles S. Fuchs, Edward L. Giovannucci

**Affiliations:** 1 Division of Medical Oncology, Department of Internal Medicine, The Ohio State University College of Medicine, 410 West 12th Avenue, 302B Wiseman Hall/CCC, Columbus, OH 43210 USA; 2 The Ohio State University Comprehensive Cancer Center - Arthur G. James Cancer Hospital and Richard J. Solove Research Institute, Columbus, OH USA; 3 Department of Nutrition, Harvard T.H. Chan School of Public Health, Boston, MA USA; 4 Department of Epidemiology, Harvard T.H. Chan School of Public Health, Boston, MA USA; 5 Harvard Medical School, Boston, MA USA; 6 Clinical and Translational Epidemiology Unit and Department of Gastroenterology, Massachusetts General Hospital, Boston, MA USA; 7 Department of Chronic Disease Epidemiology, Yale School of Public Health, New Haven, CT USA; 8 Department of Medical Oncology, Dana-Farber Cancer Institute, Boston, MA USA; 9 Department of Medicine, Yale School of Medicine and Smilow Cancer Hospital, New Haven, CT USA

**Keywords:** Colorectal cancer survival, Insulinemic dietary patterns, Insulin, C-peptide

## Abstract

**Background:**

The empirical dietary index for hyperinsulinemia (EDIH) score is a validated food-based dietary score that assesses the ability of whole-food diets to predict plasma c-peptide concentrations. Although the EDIH has been extensively applied and found to be predictive of risk of developing major chronic diseases, its influence on cancer survival has not been evaluated. We applied the EDIH score in a large cohort of colorectal cancer patients to assess the insulinemic potential of their dietary patterns after diagnosis and determine its influence on survival outcomes.

**Methods:**

We calculated EDIH scores to assess the insulinemic potential of post-diagnosis dietary patterns and examined survival outcomes in a sample of 1718 stage I-III colorectal cancer patients in the Nurses’ Health Study and Health Professionals Follow-up Study cohorts. Multivariable-adjusted Cox regression was applied to compute hazard ratios (HR) and 95% confidence intervals (CI) for colorectal cancer-specific mortality and all-cause mortality. We also examined the influence of change in diet from pre- to post-diagnosis period, on mortality.

**Results:**

During a median follow-up of 9.9 years, there were 1008 deaths, which included 272 colorectal cancer-specific deaths (27%). In the multivariable-adjusted analyses, colorectal cancer patients in the highest compared to lowest EDIH quintile, had a 66% greater risk of dying from colorectal cancer: HR, 1.66; 95% CI, 1.03, 2.69; and a 24% greater risk of all-cause death: HR, 1.24; 95%CI, 0.97, 1.58. Compared to patients who consumed low insulinemic diets from pre- to post-diagnosis period, patients who persistently consumed hyperinsulinemic diets were at higher risk of colorectal cancer death (HR,1.51; 95%CI, 0.98, 2.32) and all-cause death (HR, 1.31; 95%CI, 1.04, 2.64).

**Conclusion:**

Our findings suggest that a hyperinsulinemic dietary pattern after diagnosis of colorectal cancer is associated with poorer survival. Interventions with dietary patterns to reduce insulinemic activity and impact survivorship are warranted.

## Background

Colorectal cancer is the fourth most commonly diagnosed cancer in the United States [1]. While there is high potential for dietary patterns as a modifiable risk factor for colorectal cancer development [2, 3], very limited evidence exists among colorectal cancer survivors [4]. For example, in a recent review, we identified 50 articles published up to 2016 that reported on the association between dietary patterns and colorectal cancer development [3] but only about five articles on the association between dietary patterns and outcomes among colorectal cancer survivors [4–9]. The evidence showed that the Western dietary pattern, often characterized by high intakes of refined grains, red and processed meats, desserts, and potatoes, is associated with higher risk of all-cause mortality, but generally not with colorectal cancer-specific mortality in patients with colorectal cancer. The prudent dietary pattern, often characterized by high intakes of fruits, vegetables, whole grains, and poultry, showed similar results, with inverse associations for all-cause mortality but no consistent association with colorectal cancer-specific mortality [5–9]. Higher adherence to other dietary patterns including the Mediterranean diet score, dietary approaches to stop hypertension meal plan, American Cancer Society cancer prevention guidelines score, healthy eating index score, were generally associated with lower risk of all-cause mortality, but the associations were inconsistent across studies [5, 6, 8, 9].

Further research is therefore needed to clarify if dietary patterns are important for colorectal cancer prognosis and if dietary changes can maximally impact overall and cancer-specific survival. Biomarker-based dietary patterns may be helpful in this regard. For example, hyperinsulinemia and insulin resistance are considered important underlying mechanisms linking poor dietary patterns and lifestyle behaviors, to the development of multiple chronic diseases, including colorectal cancer [10–12]. Studies have shown positive associations between circulating c-peptide concentrations (a marker of beta cell secretory activity) and colorectal cancer risk and prognosis [13–16]. Therefore, a dietary pattern associated with hyperinsulinemia may be more predictive of outcomes following colorectal cancer diagnosis, than a dietary pattern not associated with this pathway. We previously derived the empirical dietary index for hyperinsulinemia (EDIH) score, to assess the potential of dietary patterns to influence insulinemia [17], which has been extensively applied in large cohort studies and found to be predictive of non-fasting c-peptide concentrations [17, 18], long-term weight gain [19], risk of developing colorectal cancer [20], other digestive system cancers [21], and other cancers [22]. However, the influence of dietary insulinemic potential on cancer survival outcomes has not yet been evaluated. The objective of the current study is to apply the EDIH score in a large cohort of colorectal cancer patients to assess the insulinemic potential of their dietary patterns after diagnosis and determine its influence on survival outcomes.

## Methods

### Study population

**Fig. 1:**
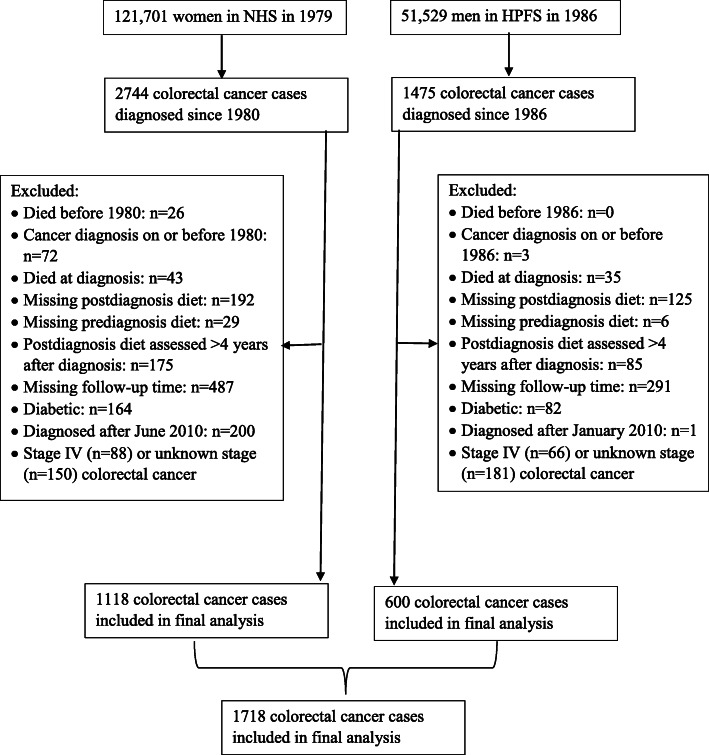
Flow chart describing the flow of participants from the full cohorts to the final analytic sample in the Nurses’ Health Study (NHS) and Health Professionals Follow-up Study (HPFS)

We used data from the Nurses’ Health Study (NHS) and the Health Professionals Follow-up Study (HPFS) - two ongoing cohorts in the United States. HPFS was initiated in 1986 and enrolled 51,529 male health professionals between the ages of 40 and 75 years [23]. NHS, initiated in 1976, enrolled 21,701 registered female nurses aged 30 to 55 years [24] (Fig. 1). Data on medical, lifestyle, and other health-related factors was collected at baseline and have been updated every 2 years thereafter. Ethical approval for our study was provided by the Harvard T.H. Chan School of Public Health, and those of participating registries as required and the institutional review boards of the Brigham and Women’s Hospital. Study participants provided consent by completing and submitting study questionnaires. Participants were free to terminate participation in the study at any time.

### Assessment of diet and the empirical dietary index for hyperinsulinemia (EDIH) score

In both cohorts, diet was assessed using a validated self-administered food frequency questionnaire (FFQ) that assessed how often, on average, participants consumed a standard portion size of various foods in the past year. In the NHS, diet was assessed in 1980, 1984, 1986 and every 4 years thereafter, whereas in the HPFS diet was assessed in 1986 and every 4 years thereafter [25]. The EDIH score, developed to empirically measure the insulinemic potential of whole diets using food groups, has been described in detail [17]. Briefly, thirty-nine food groups [26] were entered into stepwise linear regression models to identify a dietary pattern most predictive of plasma c-peptide levels. The EDIH score represents a weighted sum of 18 food groups, with higher scores indicating hyperinsulinemic diets (hyperinsulinemia) and lower scores indicating low insulinemic diets. The food groups contributing to lower EDIH scores are: wine, coffee, full-fat dairy products, whole fruit and green leafy vegetables; whereas the food groups contributing to high EDIH scores are: low-fat dairy products, French fries, low-energy beverages, cream soups, processed meat, red meat, margarine, poultry, non-dark fish, high- tomatoes, energy beverage and eggs [17].

In the current study, we calculated EDIH scores for each participant based on the self-administered FFQs. Post-diagnosis EDIH score was calculated based on the first FFQ returned at least 6 months but not more than 4 years after colorectal cancer diagnosis, thus avoiding diet assessment during active cancer therapy. The median time from diagnosis to post-diagnosis diet assessment was 2.1 years. Pre-diagnosis EDIH score was calculated based on the cumulative average of EDIH scores up to the last diet assessment before colorectal cancer diagnosis. The median time from pre-diagnosis diet assessment to diagnosis was 1.9 years.

### Patients with colorectal cancer and mortality assessment

When a colorectal cancer diagnosis was reported during the previous 2 years on the follow-up biennial questionnaires, we requested permission to obtain hospital records and pathology reports. Blinded study physicians then reviewed these records and recorded data on tumor characteristics. For non-respondents, the National Death Index was used to identify deaths and ascertain any diagnosis of colorectal cancer that contributed to death. After 30 years of follow-up for disease diagnoses (1980–2010 in NHS and 1986 to 2010 in HPFS), we identified 4219 patients with pathologically confirmed colorectal cancer. We excluded participants who died before 1980 in NHS or 1986 in HPFS, had reported any cancer (except nonmelanoma skin cancer) before colorectal cancer diagnosis, who died at diagnosis, who did not have pre-diagnosis diet or post-diagnosis diet, patients who did not complete a diet assessment between 6 months and 4 years after diagnosis or had diet assessed outside of this period, who had diabetes at colorectal cancer diagnosis, and patients with stage IV or unknown stage at diagnosis. Therefore, the current analysis included 1718 patients with stage I, II or III colorectal cancer, including 600 participants from HPFS and 1118 from NHS (Fig. 1). Deaths were ascertained through reporting by family. For persistent non-responders, we queried the National Death Index with their names, up to June 2014 for NHS and January 2014 for HPFS [27]. Cause of death was assigned by blinded physicians.

### Covariate assessment

Both cohorts assessed covariate data (e.g., medical history, lifestyle and health factors) through self-administered questionnaires every 2 years. These factors included physical activity, smoking habits, alcohol intake, multivitamin use, endoscopy status, regular use of aspirin and other non-steroidal anti-inflammatory drugs (NSAIDs), family history of colorectal cancer, weight, height, menopausal status and postmenopausal hormone use (only for women), in both cohorts as previously described. Diet assessment was conducted every 4 years [26, 28, 29].

### Statistical analysis

We categorized the EDIH score into quintiles, with cohort-specific cutoffs, then pooled the data for analysis. Person-time of follow-up was calculated from the date of post-diagnosis diet assessment to death or to last follow-up date (January 2014 in HPFS or June 2014 in NHS), whichever was first. We used the Kaplan-Meier method to generate survival curves by quintiles of EDIH score, and tested group differences (highest vs. lowest quintile) using the log-rank test. For this test the EDIH score was adjusted for total energy intake and BMI using the residual method.

Cox proportional hazards regression was used to calculate hazard ratios (HRs) of colorectal cancer-specific death or all-cause death in EDIH quintiles. Quintile cutpoints were created separately by sex and applied in the pooled sample. Given that participants must survive from diagnosis until post-diagnosis diet assessment, we used time since diagnosis as the underlying time scale to account for left truncation due to staggered entry. The Cox models were tested for the assumption of proportionality using time*covariate interaction terms and stratified by age, sex and stage. We fitted two models to the data as follows: model 1 (minimally adjusted model) included BMI, demographic factors (sex, age at diagnosis), and tumor characteristics (stage, subsite within the colon, grade of tumor differentiation). Model 2 (fully adjusted model) included all the covariates in model 1 and post-diagnosis lifestyle factors: pack-years of smoking, physical activity, regular aspirin use, pre- to post-diagnosis weight change, total alcohol intake, and pre-diagnosis dietary pattern (EDIH score). Test for linear trend of risk across EDIH quintiles was performed using the median post-diagnosis EDIH score in each EDIH quintile as a continuous variable in the Cox regression models and interpreting the *p*-value of this variable as the p-value for linear trend.

To determine how changes in the insulinemic potential of diet before and after diagnosis influence survival, we dichotomized pre and post-diagnosis EDIH scores at the median and used to create a change variable with low indicating a score below the median and high, a score above the median: Low-Low: consistently low dietary insulinemic potential before and after diagnosis (i.e., both scores below the median); Low-High: patients consuming low insulinemic diets before diagnosis and more hyperinsulinemic diets after diagnosis; High-Low: patients consuming hyperinsulinemic diets before diagnosis and then changed towards low insulinemic diets after diagnosis and High-High: patients who consistently consumed hyperinsulinemic dietary patterns before and after diagnosis. We then applied these dietary pattern changes in multivariable-adjusted Cox models to examine risk of death from colorectal cancer and from other causes.

We conducted exploratory subgroup analyses in categories of the following potential effect modifiers: sex, weight change pre- and post-diagnosis, and pre-diagnosis EDIH score. We categorized pre-diagnosis EDIH at the median (< median and ≥ median). Weight change was calculated by subtracting pre-diagnosis weight from post-diagnosis weight, the continuous weight change variable was categorized as follows: those who gained more than 2.5 kg, had a stable weight (− 2.5 kg to 2.5 kg) or lost more than 2.5 kg. We also conducted subgroup analyses by time since diagnosis (< 5 years, ≥5 years) and age at diagnosis (< 65 years, ≥65 years). Tests of interaction between post-diagnosis EDIH score and the potential effect modifiers were assessed by entering in the model the cross product of post-diagnosis EDIH score and the stratification variable and evaluated by the Wald test. All analyses were performed using SAS 9.4 for Unix. All *p*-values were two sided.

## Results

Characteristics of patients (65.1% women) with colorectal cancer after diagnosis are shown in Table 1. Mean age at diagnosis was 68.3 years and mean post-diagnosis BMI was 26.1 kg/m^2^, with 58.5% of patients classified as overweight or obese. Regarding disease stage, 71.5% had stage I or II and 28.5% had stage III disease. During a median follow-up of 9.9 years, there were 1008 all-cause deaths, which included 272 colorectal cancer-specific deaths. Median overall survival by cancer stage was 10.7 years for those with stage I disease, 9.9 years for those with stage II and 8.0 years for those with stage III disease. Forty-one percent of patients maintained a stable weight between − 2.5 kg and + 2.5 kg between the pre-diagnosis and post-diagnosis period, while 34% lost more than 2.5 kg body weight and 25% gained 2.5 kg body weight in the same period. Colorectal cancer patients with the most hyperinsulinemic dietary patterns after diagnosis (quintile 5) tended to have higher body weight and lower physical activity. For example, the average BMI among those classified in quintile 5 was 27.3 kg/m^2^ and the average physical activity was 13.8 MET-hour/week compared with 25.1 kg/m^2^ and 21.8 MET-hour/week among those in quintile 1. Also, patients consuming the most hyperinsulinemic dietary patterns were less likely to have stage I disease and they experienced shorter survival times (Table 1).

**Table 1:** Postdiagnosis characteristics of colorectal cancer patients by quintiles of postdiagnosis empirical dietary index for hyperinsulinemia (EDIH) score; *n* = 1718

Characteristic	Total population (*n* = 1718)	Quintiles of the empirical dietary index for hyperinsulinemia (EDIH) score^a,b^				
		Quintile 1 (−4.28 to < −0.71) (*n* = 344)	Quintile 2 (−0.71 to < − 0.25) (*n* = 353)	Quintile 3 (− 0.25 to < 0.10) (*n* = 349)	Quintile 4 (0.10 to < 0.51) (*n* = 336)	Quintile 5 (0.51 to 4.00) (*n* = 336)
Female, %	65.1	65.1	65.1	65.1	65.1	65.1
Age at diagnosis^d^	68.3 (9.2)	68.1 (8.8)	69.1 (8.8)	68.9 (8.8)	67.6 (9.6)	67.6 (9.8)
Age at diagnosis by sex^d^						
Female	67.6 (9.0)	68.0 (8.7)	68.3 (8.6)	68.1 (8.6)	67.3 (9.5)	66.0 (9.6)
Male	69.7 (9.3)	68.4 (9.2)	70.6 (8.9)	70.4 (8.9)	68.2 (9.7)	70.6 (9.7)
Current smoker, %	7.1	4.6	6.6	9.0	6.4	9.0
Pack-years of smoking	16.3 (22.2)	16.0 (20.8)	16.0 (20.5)	14.1 (22.0)	17.8 (23.9)	17.7 (23.4)
Body mass index, kg/m^2^	26.1 (4.4)	25.1 (4.0)	25.6 (4.1)	25.9 (4.6)	26.5 (4.5)	27.3 (4.6)
Overweight (BMI ≥ 25, < 30), %	42.5	35.7	41.5	42.7	47.4	45.0
Obese (BMI ≥ 30), %	16.0	10.4	13.0	14.3	18.1	25.0
Physical activity, MET-h/week^c^	18.5 (22.5)	21.8 (25.4)	20.5 (24.3)	18.1 (23.3)	18.2 (20.7)	13.8 (17.7)
Physical activity, MET-h/week^c,d^ by sex						
Female	15.1 (19.8)	17.4 (19.9)	17.4 (23.5)	14.1 (20.8)	14.5 (19.0)	11.9 (13.5)
Male	25.0 (25.7)	30.4 (31.3)	26.2 (24.7)	25.3 (24.4)	25.8 (23.1)	17.1 (22.6)
Non-alcohol drinkers, %	40.2	24.3	38.3	39.1	46.3	53.0
Regular aspirin use, %	30.4	30.7	30.8	31.2	26.6	33.0
Location of cancer in the colon, %						
Proximal colon	43.7	45.4	45.7	41.1	43.7	42.6
Distal colon	33.3	33.1	31.2	31.5	37.0	32.9
Rectum	22.3	21.0	22.0	26.7	17.9	24.2
Unspecified	0.7	0.5	1.1	0.8	1.4	0.2
Stage at diagnosis, %						
Stage I	37.7	39.9	36.0	35.1	39.7	36.1
Stage II	33.8	32.2	35.7	35.5	31.4	34.9
Stage III	28.5	27.9	28.3	29.3	29.0	29.0
Median survival time, years	10.8 (7.3)	11.5 (7.3)	11.2 (7.7)	11.0 (7.2)	10.1 (7.2)	10.2 (6.9)
Median survival time by stage,^d^ years						
Stage I	10.7	13.0	11.6	11.7	8.6	9.9
Stage II	9.9	9.8	9.9	9.9	9.1	10.6
Stage III	8.0	9.2	8.2	8.5	10.0	6.1
Weight change categories^d^, %						
Stable weight (−2.5 to 2.5 kg)	41.6	47.5	41.0	39.5	40.4	38.4
Gained more than 2.5 kg	24.7	19.8	28.9	25.1	22.4	26.6
Lost more than 2.5 kg	33.7	32.7	30.1	35.4	37.2	34.9

^a^Values are means (SD) for continuous variables and percentages for categorical variables and are standardized to the age distribution of the study population

^b^EDIH scores were adjusted for total energy intake

^c^Metabolic equivalents from recreational and leisure-time activities

^d^Value is not age adjusted

Patients consuming low insulinemic dietary patterns had higher intakes of wholegrains, nuts, vegetables, whole fruits and coffee; and lower intakes of refined grains, cream soup, eggs, French fries, butter, margarine, sugar-sweetened beverages, red meat and processed meat. In terms of the nutrient profile resulting from this post-diagnosis dietary pattern, patients consuming a low insulinemic dietary pattern had higher intakes of total carbohydrates and total fiber and lower intakes of total fat, total protein and branched-chain amino acids (Table 2).

**Table 2:** Median (5th, 95th percentile) food and nutrient intake profiles of colorectal cancer patients by quintiles of post-diagnosis empirical dietary index for hyperinsulinemia (EDIH) score

	Total population (*n* = 1718)	Quintiles of the empirical dietary index for hyperinsulinemia (EDIH) score^a,b^				
		Quintile 1 (*n* = 344)	Quintile 2 (*n* = 353)	Quintile 3 (*n* = 349)	Quintile 4 (*n* = 336)	Quintile 5 (*n* = 336)
**Foods, servings/week**						
Processed meat	1 (0, 5)	0.5 (0, 3.6)	1 (0, 4)	1 (0, 4)	1.1 (0, 6)	1.7 (0, 8.5)
Red meat	2 (0, 7.6)	1.5 (0, 5.1)	1.7 (0, 6.5)	2 (0, 6)	2.5 (0.5, 9)	3.9 (0.5, 10)
High-energy sugary beverages	0.5 (0, 7.1)	0 (0, 3.9)	0.5 (0, 4.1)	0.5 (0, 7)	0.6 (0, 7.6)	0.8 (0, 17.5)
Low-energy sugary beverages	0.5 (0, 21)	0 (0, 7.6)	0 (0, 7.5)	0.5 (0, 17.5)	1 (0, 18.6)	1.5 (0, 70)
Margarine	1 (0, 17.5)	0.5 (0, 17.5)	1 (0, 17.5)	1 (0, 17.5)	3 (0, 17.5)	3 (0, 17.5)
Butter	0 (0, 17.5)	0 (0, 7)	0 (0, 7)	0 (0, 7)	0 (0, 7)	0.5 (0, 17.5)
French fries	0 (0, 1)	0 (0, 1)	0 (0, 1)	0 (0, 1)	0.5 (0, 1)	0.5 (0, 3)
Non-dark fish	1.1 (0, 4.5)	1.1 (0, 5)	1 (0, 4.5)	1.5 (0, 4.1)	1.5 (0, 4.1)	1.5 (0, 4.5)
Eggs	1 (0, 5.5)	1 (0, 3)	1 (0, 3)	1 (0, 3)	1 (0, 5.5)	3 (0, 7)
Low-fat dairy	2 (0, 18.5)	3 (0, 19)	3 (0, 18.1)	2.5 (0, 18.6)	2.2 (0, 14.5)	1.5 (0, 18.5)
Cream soup	0 (0, 1)	0 (0, 1)	0 (0, 1)	0 (0, 1)	0.5 (0, 1)	0.5 (0, 3)
Refined grains	6.4 (0, 25)	6 (0, 23.5)	5.7 (0.5, 26.5)	5.5 (0, 19.5)	7.0 (0, 24.9)	7.5 (0, 26.2)
Tomato	4.0 (0.6, 10.5)	4 (0.6, 10)	3.6 (0.5, 10)	4 (0.6, 10)	4.1 (1, 10.5)	4 (1, 12)
Poultry	1.1 (0, 5.5)	1 (0, 5.5)	1 (0, 5.5)	1 (0, 4)	1.5 (0, 5.5)	1.5 (0, 6)
Dark fish	1.5 (0, 5.5)	1.7 (0, 6)	1.1 (0, 5.5)	1.5 (0, 4.6)	1.5 (0, 5)	1.5 (0, 6.7)
Full-fat dairy	4.0 (0.5, 17.5)	4.5 (0, 23)	3.6 (0.5, 13)	4 (0, 14.6)	4.0 (0.5, 14.5)	4 (0.5, 14)
Coffee	7.5 (0, 35)	17.5 (0, 42)	14 (0, 38)	7 (0, 35)	7 (0, 35)	7 (0, 35)
Tea	1 (0, 31.5)	1 (0, 29)	3 (0, 24)	1 (0, 31.5)	1 (0, 21.6)	1 (0, 42.6)
Whole fruit	10 (1.5, 28.6)	14 (2.5, 36.5)	11.3 (1.7, 25.7)	9.4 (1.5, 24)	9.5 (1.5, 22)	7.4 (1, 28.1
Fruit juice	3.5 (0, 18.1)	4 (0, 21)	3 (0, 17.5)	3.5 (0, 17.5)	3.5 (0, 14.1)	3 (0, 28)
Potatoes	3.0 (0, 7)	3 (0, 14)	3 (0, 7)	3 (0.5, 7)	3 (0.5, 7)	3 (0, 14)
Green-leafy vegetables	4 (0.5, 12.5)	6 (1, 15)	4 (0.5, 11.6)	3.6 (0.5, 12.7)	3.5 (0.5, 11)	3 (0.5, 10)
Dark-yellow vegetables	2 (0, 8)	2.9 (0, 9)	2.1 (0, 9)	2 (0, 8)	2 (0.5, 7.1)	1.7 (0, 7.1)
Other vegetables	7.5 (1, 32.6)	9.1 (1, 34.3)	7.3 (1, 31.2)	7.4 (1.1, 32.6)	6.4 (1, 29.2)	6.9 (1.0, 38.6)
Nuts	1.5 (0, 9)	1.5 (0, 14)	1.5 (0, 9)	1.1 (0, 8.5)	1.5 (0, 9)	1.1 (0, 8)
Total alcohol intake, drinks/week	1 (0, 18.6)	4 (0, 24.5)	1 (0, 18)	1 (0, 18)	0.5 (0, 18.1)	0 (0, 17.5)
Whole grains	6.6 (0, 24.5)	8.1 (0, 29)	7 (0, 25)	7 (0, 21)	5.6 (0, 23)	4 (0, 22.5)
**Nutrient profile**						
Total carbohydrates, g/d	223 (163, 297)	229 (166, 313)	230 (167, 301)	226 (165, 298)	219 (165, 283)	215 (149, 280)
Total protein, g/d	73 (49, 102)	70 (48, 96)	73 (49, 99)	72 (51, 99)	75 (53, 100)	76 (49, 114)
Branched-chain amino acids, g/d	12.7 (8.4, 18)	12.2 (7.9, 16.8)	12.6 (8.4, 17.6)	12.5 (8.6, 17.5)	13.0 (8.9, 17.7)	13.4 (8.2, 19.5)
Total fat, g/d	58 (36, 83)	53 (32, 78)	56 (35, 77)	58 (39, 82)	59 (39, 84)	63 (40, 91)
Total fiber, g/d	20.1 (11.8, 32.6)	22.5 (13.1, 35.7)	21.5 (13.6, 33.6)	19.6 (12.1, 32.7)	19.3 (11.7, 29)	18.3 (10.1, 29.3)

^a^Values are means (SD) for continuous variables and percentages for categorical variables and are standardized to the age distribution of the study population

^b^EDIH scores were adjusted for total energy intake

**Fig. 2:**
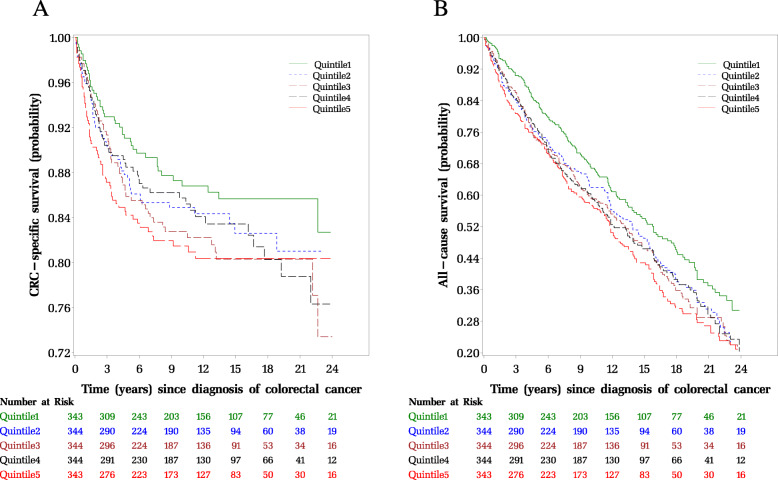
Kaplan–Meier curves of (**a**) colorectal cancer-specific and (**b**) overall survival among patients with colorectal cancer by quintile of post-diagnosis empirical dietary index for hyperinsulinemia (EDIH) score. Log-rank *p*-values were calculated to test group differences (quintile 1 vs. 5) and adjusted for post-diagnosis total energy intake and post-diagnosis body mass index

Kaplan-Meier curves by quintiles of EDIH score are shown in Fig. 2, with patients consuming a low insulinemic diet (quintile 1) experiencing better survival for colorectal cancer-specific and overall mortality, compared to those consuming hyperinsulinemic diets (quintile 5). In the multivariable-adjusted analyses, we found that a hyperinsulinemic post-diagnosis dietary pattern was associated with higher risk of colorectal cancer-specific mortality and all-cause mortality (Table 3). Comparing colorectal cancer patients classified in the highest EDIH quintile to those in the lowest quintile, there was a 66% higher risk of colorectal cancer-specific death: HR, 1.66; 95%CI, 1.03, 2.69; P-trend = 0.07; and a 24% higher risk of all-cause death: HR, 1.24; 95%CI, 0.97, 1.58; P-trend = 0.08, after accounting for pre-diagnosis dietary insulinemic potential among other confounding variables (Table 3).

**Table 3:** Hazard ratios (95% CI) for colorectal cancer-specific and all-cause mortality among patients with colorectal cancer by quintile of post-diagnosis EDIH score

	Quintiles of the empirical dietary index for hyperinsulinemia (EDIH) score					P-trend
Statistical model	Quintile 1	Quintile 2	Quintile 3	Quintile 4	Quintile 5	
**Colorectal cancer-specific mortality**						
Deaths/Patients alive	43/300	60/284	56/288	57/287	56/287	
Minimally-adjusted model	1 (reference)	1.39 (0.94, 20.6)	1.27 (0.85, 1.90)	1.41 (0.94, 2.11)	1.62 (1.08, 2.42)	0.03
Fully adjusted model	1 (reference)	1.44 (0.96, 2.18)	1.33 (0.87, 2.02)	1.45 (0.94, 2.26)	1.66 (1.03, 2.69)	0.07
**All-cause mortality**						
Deaths/Patients alive	178/165	204/140	204/140	215/129	207/136	
Minimally-adjusted model	1 (reference)	1.22 (1.00, 1.49)	1.21 (0.99, 1.48)	1.46 (1.19, 1.78)	1.35 (1.10, 1.65)	0.002
Fully adjusted model	1 (reference)	1.23 (1.00, 1.52)	1.20 (0.97, 1.49)	1.39 (1.11, 1.73)	1.24 (0.97, 1.58)	0.08

The minimally-adjusted models was adjusted for age at diagnosis, post-diagnosis body mass index, total energy intake, sex, race, year of diagnosis, cancer stage, grade of tumor differentiation, and location of primary tumor within the colon; the fully-adjusted model was additionally adjusted for post-diagnosis physical activity, post-diagnosis pack years of smoking, post-diagnosis regular aspirin use, weight change pre to post-diagnosis, post-diagnosis total alcohol intake, and pre-diagnosis EDIH score

**Fig. 3:**
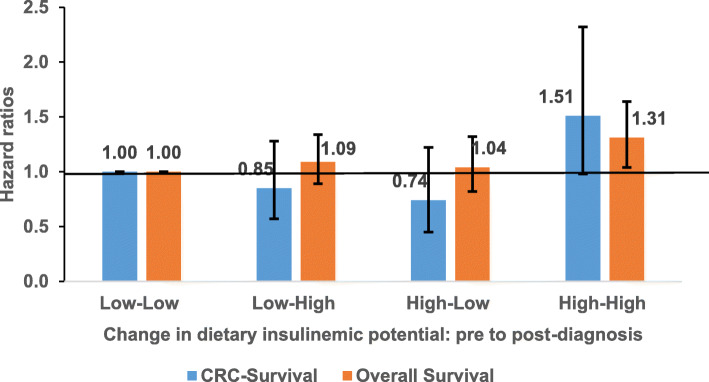
Hazard ratios for the association of change in dietary insulinemic potential between pre-diagnosis diet and post-diagnosis diet and risk of dying form colorectal cancer (CRC-survival) and from all causes combined (overall survival). EDIH scores were dichotomized at the median: Low-Low, the reference category, represents participants who persistently consumed low insulinemic diets (below the median EDIH) from pre to post-diagnosis period; Low-High are those who changed from low insulinemic diets towards more hyperinsulinemic diets; High-Low represents those who changed from consuming hyperinsulinemic diets prior to diagnosis towards consuming low insulinemic diets after diagnosis, whereas High-High represents those who persistently consumed hyperinsulinemic diets prior to diagnosis and after diagnosis. The number of deaths / patients alive in the four categories were as follows: CRC-survival: Low-Low 97/541, Low-High 33/196, High-Low 30/205, High-High 112/504; Overall survival: Low-Low 347/291, Low-High 140/89, High-Low 139/96, High-High 382/234. Models were adjusted for age at diagnosis, post-diagnosis body mass index, total energy intake, sex, race, year of diagnosis, cancer stage, grade of tumor differentiation, location of primary tumor within the colon, post-diagnosis physical activity, post-diagnosis pack years of smoking, post-diagnosis regular aspirin use, weight change pre to post-diagnosis, post-diagnosis total alcohol intake, and pre-diagnosis EDIH score

In relation to changes in the insulinemic potential of the diet before and after diagnosis, patients who consumed a more hyperinsulinemic dietary pattern consistently before and after diagnosis were at higher risk of dying from colorectal cancer (HR, 1.51; 95% CI, 0.98, 2.32) and from other causes (HR, 1.31; 95% CI, 1.04, 1.64), compared to patients who consistently consumed a low insulinemic dietary pattern before and after diagnosis (Fig. 3).

In subgroups of potential effect modifiers, risk of colorectal cancer-specific mortality was significantly elevated among women, and among those who lost body weight, those who were consuming a hyperinsulinemic dietary pattern before diagnosis and those younger than 65 years. For these subgroup analyses, interactions were statistically significant only for sex in all-cause mortality (Table 4).

**Table 4:** Subgroup analyses of the association between dietary insulinemic potential and colorectal cancer-specific and all-cause mortality

Subgroup	Deaths/Patients alive	EDIH quintiles				P-trend	P-interaction
		Quartile 1	Quartile 2	Quartile 3	Quartile 4		
**Colorectal cancer-specific mortality**							
Sex							0.48
Men	83/517	1 (ref)	1.15 (0.60, 2.18)	1.09 (0.56, 2.12)	1.02 (0.50, 2.08)	0.98	
Women	189/929	1 (ref)	1.19 (0.76, 1.89)	1.41 (0.90, 2.21)	1.71 (1.01, 2.88)	0.03	
Weight change (post minus pre-diagnosis weight)							0.18
Stable weight (−2.5 to 2.5 kg)	98/616	1 (ref)	1.13 (0.63, 2.05)	1.37 (0.76, 2.48)	1.04 (0.51, 2.13)	0.73	
Weight gain > 2.5 kg	66/359	1 (ref)	1.01 (0.50, 2.02)	0.99 (0.46, 2.14)	1.18 (0.50, 2.80)	0.73	
Weight loss > 2.5 kg	108/471	1 (ref)	1.38 (0.70, 2.32)	1.91 (0.99, 3.68)	2.76 (1.32, 5.60)	0.003	
Pre-diagnosis EDIH score							0.06
< Median	130/737	1 (ref)	1.19 (0.77, 1.83)	0.86 (0.51, 1.45)	0.92 (0.45, 1.87)	0.66	
≥ Median	142/708	1 (ref)	1.19 (0.52, 2.71)	2.00 (0.95, 4.24)	2.13 (0.99, 4.58)	0.01	
Age group at diagnosis							0.54
< 65 years	107/487	1 (ref)	0.89 (0.49, 1.62)	1.03 (0.58, 1.83)	1.39 (0.73, 2.64)	0.29	
≥ 65 years	165/959	1 (ref)	1.29 (0.80, 2.07)	1.58 (0.97, 2.58)	1.32 (0.76, 2.29)	0.28	
Time since diagnosis							0.41
< 5 years	207/221	1 (ref)	0.91 (0.58, 1.43)	0.98 (0.63, 1.52)	1.40 (0.86, 2.30)	0.16	
≥ 5 years	65/1225	1 (ref)	0.97 (0.47, 2.01)	1.08 (0.53, 2.23)	0.56 (0.22, 1.38)	0.26	
**All-cause mortality**							
Sex							0.001
Men	398/202	1 (ref)	1.13 (0.85, 1.52)	0.84 (0.61, 1.16)	0.90 (0.65, 1.25)	0.36	
Women	610/508	1 (ref)	1.23 (0.96, 1.57)	1.62 (1.27, 2.08)	1.72 (1.29, 2.31)	< 0.0001	
Weight change (post minus prediagnosis weight)							0.57
Stable weight (−2.5 to 2.5 kg)	379/335	1 (ref)	1.35 (1.00, 1.81)	1.46 (1.07, 2.00)	1.30 (0.91, 1.86)	0.13	
Weight gain > 2.5 kg	241/184	1 (ref)	0.85 (0.57, 1.28)	1.09 (0.72, 1.66)	1.28 (0.80, 2.06)	0.18	
Weight loss > 2.5 kg	388/191	1 (ref)	1.22 (0.89, 1.67)	1.30 (0.94, 1.80)	1.57 (1.10, 2.25)	0.01	
Prediagnosis EDIH score							0.16
< Median	487/380	1 (ref)	1.27 (1.01, 1.59)	1.12 (0.85, 1.48)	1.42 (1.02, 1.96)	0.05	
≥ Median	521/330	1 (ref)	1.15 (0.79, 1.66)	1.48 (1.06, 2.08)	1.36 (0.96, 1.93)	0.07	
Age group at diagnosis							0.75
< 65 years	265/329	1 (ref)	1.10 (0.74, 1.63)	1.41 (0.96, 2.05)	1.67 (1.10, 2.52)	0.009	
≥ 65 years	743/381	1 (ref)	1.16 (0.94, 1.44)	1.26 (1,00, 1.58)	1.22 (0.94, 1.57)	0.12	
Time since diagnosis							0.79
< 5 years	394/34	1 (ref)	1.09 (0.79, 1.48)	1.18 (0.86, 1.62)	1.34 (0.94, 1.93)	0.08	
≥ 5 years	614/676	1 (ref)	1.10 (0.87, 1.39)	1.22 (0.95, 1.56)	1.26 (0.95, 1.66)	0.09	

Models were adjusted for age at diagnosis, postdiagnosis body mass index, total energy intake, sex, race, year of diagnosis, cancer stage, grade of tumor differentiation, and location of primary tumor within the colon, postdiagnosis physical activity, postdiagnosis pack years of smoking, postdiagnosis regular aspirin use, weight change pre to postdiagnosis and postdiagnosis total alcohol intake, and pre-diagnosis EDIH score

## Discussion

In the current study, we showed that habitual consumption of hyperinsulinemic dietary patterns after colorectal cancer diagnosis, or consumption of a hyperinsulinemic dietary pattern consistently before and after diagnosis, was associated with higher risk of dying from colorectal cancer and from all causes combined.

The insulinemic potential of diet was first estimated by the insulin index [30], which is based on a concept similar to the more widely used glycemic index, that characterizes carbohydrate-containing foods according to their ability to raise blood glucose concentrations postprandially compared with a reference food (glucose or white bread) [31]. Though carbohydrate content is one important factor influencing insulin response, foods can also stimulate insulin secretion in a carbohydrate-independent manner. The insulin index directly quantifies the postprandial insulinemic potential of a food, and takes into account foods with a low or no carbohydrate content [30]. It is important to understand that the insulin index, which was used in most previous studies of dietary insulinemic potential and colorectal cancer survival, is conceptually and technically different from the EDIH, and essentially uncorrelated (Spearman *r* = − 0.03). The principle of the insulin index is how a particular food item stimulated insulin secretion independent of underlying insulin resistance, whereas the EDIH is primarily driven by insulin resistance. For colorectal cancer, the only other paper using the EDIH was on cancer incidence [20].

Both the insulin index and glycemic index assess the postprandial (short-term) effects of the diet, unlike the EDIH score which predicts integrated insulin exposure (i.e., both fasting and non-fasting), based on habitual (long-term) dietary intake [18]. Post-diagnosis insulin index and insulin load have been linked to higher risk of dying from colorectal cancer [32, 33]. Higher dietary insulin load and insulin index after diagnosis of colorectal cancer were associated with increased risk of colorectal cancer-specific and overall mortality [33]. The association of post-diagnosis glycemic indices with colorectal cancer prognosis has been inconsistent. Whereas one study found higher risk of colorectal cancer recurrence and death associated with higher glycemic load but not higher glycemic index [34]; another found no association between glycemic load or glycemic index and colorectal cancer survival [35]. Glycemic scores are primarily reflective of the postprandial glucose responses of carbohydrate-containing foods, whereas the EDIH score directly reflects insulin increases induced by components of the dietary pattern that may or may not be contributing to calories (e.g., coffee). Current study findings therefore suggest that the direct effect of the diet on insulin may be more important than the effect of diet on glucose for colorectal cancer prognosis. Though the glycemic index is a measure of the short-term (postprandial) effect of the diet on glucose concentrations, it is possible that such a habitual dietary pattern could, over time, lead to sustained hyperinsulinemia and insulin resistance, which could then mediate colorectal cancer prognosis. However, a previous study in these cohorts did not observe an association between an overall low-carbohydrate diet score and colorectal cancer or overall mortality; although those who consumed a plant-rich, low-carbohydrate diet, which emphasized plant sources of fat and protein with moderate consumption of animal products, had lower risk of colorectal cancer-specific mortality [36].

Insulin is a growth factor and major regulator of cell metabolism, and its effects in target cells are mediated by the insulin receptor, a transmembrane protein with enzymatic activity [37]. Evidence suggest that insulin stimulates growth mainly via its own receptor and not the IGF-1 receptor, and that in many cancer cells, the insulin receptor is overexpressed and the A isoform, which has a predominant mitogenic effect, is more represented than the B isoform [38]. The metabolic pathway stimulated by the activated insulin receptor to regulate glucose, protein, and lipid metabolism involves the PI3K/Akt pathway [39]. These characteristics provide a selective growth advantage to cancer cells when exposed to insulin. Therefore, all conditions of hyperinsulinemia, both endogenous (e.g., type 2 diabetes, metabolic syndrome, obesity) and exogenous (e.g., hyperinsulinemic diets; which also influence some of the endogenous conditions) [19, 40], will increase cancer risk and mortality [37].

Although interactions for most of the subgroup analyses were not statistically significant, some of the findings merit some discussion. The associations were stronger among women than among men, which may be related to several factors: the larger sample size and statistical power in our evaluation of women, potential confounding with age as women were younger on average than men, and a true biological interaction based upon endocrine and associated metabolic factors. We also observed that there were worse outcomes among patients who lost weight than among those who maintained a stable weight from pre- to post-diagnosis period, which may be consistent with complications of progressing disease leading to poor diet intake.

Major strengths of our study include the use of a food-based EDIH score that is correlated with circulating c-peptide concentrations [17, 18]. We had access to comprehensive pre- and post-diagnosis data on diet and important covariates, which reduces the potential for residual confounding and recall bias. Our findings also accounted for potential bias from staggered entry due to differences between participants in the time between diagnosis and post-diagnosis diet assessment. Limitations to be considered in interpreting our findings include: potential measurement error in the self-reported dietary and lifestyle data, though prior studies in the HPFS and NHS that evaluated the relative validity of FFQ data have shown reasonably good correlations between FFQ and diet records [23, 25, 41]. Though we adjusted for several potential confounding variables, a hyperinsulinemic dietary pattern may be associated with other factors not included in the current study. Therefore, we cannot completely rule out confounding by unmeasured variables. Given that we did not have information on cancer treatment, which could influence dietary choices of cancer patients or modify the diet and survival association, we adjusted all analyses by cancer stage at diagnosis, which is the principal determinant of colorectal cancer treatment.

## Conclusion

In this large prospective study, a higher EDIH score, reflecting higher insulinemic potential of the diet, was associated with higher risk of death from colorectal cancer and from all causes. Taken together, our results suggest that this association may be mediated partly through mechanisms involving hyperinsulinemia. Interventions with dietary patterns to reduce insulinemia may enhance survivorship among colorectal cancer patients.

## Data Availability

The datasets generated and/or analyzed during the current study reside at the Harvard T. H. Chan School of Public Health, Boston, MA, and are available from the corresponding author on reasonable request (https://docs.google.com/forms/d/e/1FAIpQLScAPV23ZIBpkk9CyEJ1OcFJjMol9elKEpLYnPu7g3PgBL57XA/viewform).

## Abbreviations

BMI: Body Mass Index
CI: Confidence interval
EDIH: Empirical Dietary Index for Hyperinsulinemia score
FFQ: Food Frequency Questionnaire
HPFS: Health Professionals Follow-up Study
HR: Hazard ratio
MET-hour/week: Metabolic Equivalent hours per week
NHS: Nurses’ Health Study
NSAIDs: Non-Steroidal Anti-inflammatory Drugs
PI3K/Akt: Phosphatidylinositol 3-kinase/Protein kinase B
SAS®: Statistical Analysis Software®
